# Polygoni Multiflori Radix Improves Dyslipidemia by Regulating Hepatocyte Lipid Metabolism Mediated via the AMPK/SREBP-2/PCSK9/LDLR Signaling Pathway

**DOI:** 10.3390/metabo16040230

**Published:** 2026-03-31

**Authors:** Tongye Wang, Lijuan Zhang, Yue Wang, Jianguo Xing, Lei Xu, Xu Hu, Wenling Su, Ruifang Zheng

**Affiliations:** 1 Xinjiang Key Laboratory of Uyghur Medical Research, Xinjiang Institute of Materia Medica, Urumqi 830004, China; ty07152022@163.com (T.W.); wangyue20165643@163.com (Y.W.); xjguodd@163.com (J.X.); xulei13579912207@163.com (L.X.); yaki_hx@163.com (X.H.); 2 School of Pharmacy, Xinjiang Medical University, Urumqi 830017, China; 3 Department of Clinical Pharmacy, School of Preclinical Medicine and Clinical Pharmacy, China Pharmaceutical University, Nanjing 211198, China

**Keywords:** *Polygonum multiflorum*, AMPK, SREBP-2, PCSK9, LDLR, signaling pathway, lipid metabolism, dyslipidemia, molecular dynamics simulation

## Abstract

Objective: To clarify the molecular mechanism of Polygoni Multiflori Radix in improving dyslipidemia and provide a scientific basis for its clinical application. Methods: Network pharmacology, molecular docking and simulation were used to predict and verify active components and core targets of Polygoni Multiflori Radix. HFD-induced hyperlipidemic mice were grouped and administered for 28 days; serum indices, hepatic pathology and AMPK/SREBP-2/PCSK9/LDLR pathway expression were detected. Results: Twenty-two active components, 101 potential targets and the AMPK pathway (core) were identified. TSG, its main component, bound stably to AMPK/SREBP-2/PCSK9/LDLR. Polygoni Multiflori Radix dose-dependently improved HFD-induced abnormalities in mice (*p* < 0.05 or *p* < 0.01). Conclusions: Polygoni Multiflori Radix effectively improves HFD-induced dyslipidemia by regulating the AMPK/SREBP-2/PCSK9/LDLR pathway to ameliorate hepatocyte lipid metabolism and reduce oxidative stress/liver injury.

## 1. Introduction

Dyslipidemia (HLP) is a metabolic disorder characterized by elevated serum levels of total cholesterol (TC), triglycerides (TG), and low-density lipoprotein cholesterol (LDL-c), as well as decreased high-density lipoprotein cholesterol (HDL-c) [1]. It is not only a core risk factor for atherosclerosis and coronary heart disease but also induces various complications including fatty liver disease and diabetes [2]. At present, statins are the main lipid-lowering drugs used clinically; however, these agents are associated with side effects such as an increased risk of new-onset diabetes [3]. Therefore, the development of safe and effective novel lipid-lowering drugs holds important clinical value.

The pathogenesis of dyslipidemia is closely related to disorders of lipid metabolism, abnormal cholesterol metabolism, and imbalanced oxidative stress [4,5,6,7]. The AMPK/SREBP-2/PCSK9/LDLR signaling pathway plays a central role in regulating the synthesis and metabolism of cholesterol, fatty acids, and TG [8,9]. Adenosine 5′-monophosphate-activated protein kinase (AMPK), known as the “master switch” of cellular energy metabolism, maintains energy homeostasis by suppressing anabolic processes and promoting catabolic pathways [10,11,12,13]. Sterol regulatory element-binding protein 2 (SREBP-2) activates the transcriptional expression of proprotein convertase subtilisin/kexin type 9 (PCSK9), which in turn promotes the degradation of hepatic low-density lipoprotein receptor (LDLR), thereby reducing the uptake of serum LDL-c by hepatocytes [14]. Nevertheless, the precise mechanisms underlying the coordinated regulation of energy metabolism and oxidative stress in dyslipidemia by AMPK and LDLR remain to be further elucidated.

As a traditional tonic Chinese herbal medicine, *Polygonum multiflorum* Thunb. (*P. multiflorum*) has long been used in the treatment of cardiovascular diseases. Clinical studies have confirmed its marked lipid-lowering and anti-atherosclerotic effects [15,16]. The major active constituents of *P. multiflorum* include 2,3,5,4′-tetrahydroxystilbene-2-O-β-D-glucoside (THSG), anthraquinones (e.g., emodin), polysaccharides, and phospholipids. Among these, THSG reduces serum TC and LDL-c levels [17], emodin ameliorates lipid metabolic disorders in hyperlipidemic models [18,19], and polysaccharides exert immunomodulatory functions [20,21]. Despite the confirmed clinical lipid-lowering efficacy of *P. multiflorum* and the preliminary exploration of its partially active components, there are still obvious research gaps that need to be filled, which constitute the core source of the research concept of this study. Previous studies have mostly focused on the single-component or single-target effects of *P. multiflorum*, lacking a systematic exploration of the overall regulatory mechanism of its lipid-lowering effect, especially the specific regulatory role and molecular mechanism of *P. multiflorum* in the AMPK/SREBP-2/PCSK9/LDLR signaling pathway (a key pathway for lipid metabolism) have not been clarified. In addition, the coordinated regulatory relationship between the active components of *P. multiflorum* and the AMPK/SREBP-2/PCSK9/LDLR signaling pathway, as well as how they jointly alleviate lipid accumulation and liver injury in dyslipidemia, remains unclear. Therefore, the innovation of this study lies in breaking through the limitations of previous single-component and single-pathway research, adopting an integrated strategy combining network pharmacology prediction, molecular docking, molecular dynamics simulation validation, and in vivo animal experiments to systematically decipher the multi-component, multi-target, and multi-pathway regulatory mechanism of *P. multiflorum* in improving dyslipidemia. This study is expected to clarify the material basis and molecular mechanism of the lipid-lowering effect of *P. multiflorum*, make up for the deficiencies of previous studies, and further provide more sufficient and in-depth scientific evidence for its clinical application, which also conforms to the research trend of developing safe and effective natural lipid-lowering drugs.

In the present study, an integrated strategy combining network pharmacology prediction, molecular docking, molecular dynamics simulation validation, and in vivo animal experiments was employed to systematically investigate the molecular mechanism by which *P. multiflorum* ameliorates dyslipidemia via regulating the AMPK/SREBP-2/PCSK9/LDLR signaling pathway, aiming to provide scientific evidence for its clinical application.

**Figure 1:**
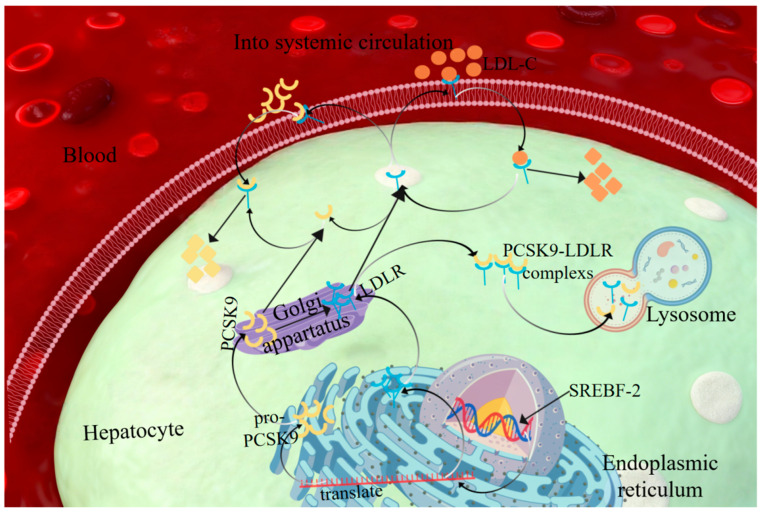
Schematic Diagram of the Mechanism by Which HSW Targets the AMPK/SREBP-2/PCSK9/LDLR Axis to Regulate Cholesterol Homeostasis. In the dyslipidemia model, HSW activates AMPK, inhibits the SREBP-2/PCSK9 pathway, and reduces the degradation of LDLR, thereby enhancing the uptake of LDL-C by hepatocytes and improving dyslipidemia.

This study aimed to systematically evaluate the lipid-lowering efficacy of *P. multiflorum* and explore its underlying mechanisms. We hypothesized that *P. multiflorum* may exert lipid-lowering effects by regulating the hepatic AMPK/SREBP-2/PCSK9/LDLR signaling pathway, thereby attenuating lipid accumulation and liver injury. A schematic diagram of the proposed mechanism is presented in Figure 1.

## 2. Materials and Methods

### 2.1. Animals

Male Kunming (KM) mice (20 ± 2 g) were purchased from Henan Skbeisi Biotechnology Co., Ltd. (SPF grade, Zhengzhou, China) with the license number SCXK (Yu) 2020-0005. The mice were housed in an SPF-grade animal facility under controlled environmental conditions: temperature of 25 ± 2 °C, relative humidity of 60% ± 10%, and a 12 h light/dark cycle, with free access to food and water. All animal experiments were approved by the Animal Ethics Committee of Xinjiang Uygur Autonomous Region Institute of Materia Medica (Ethics No.: XJIMM-20231102) and strictly followed the committee’s guidelines and the NIH Guide for the Care and Use of Laboratory Animals.

Animal welfare was emphasized throughout the experiment, and the 3R principles were strictly followed: appropriate KM mice were selected to avoid redundant use of higher animals; the minimum sample size was determined by statistical power analysis to reduce the number of animals; the housing environment and experimental procedures were optimized, and all operations were performed as standardized by professional researchers to minimize animal suffering and ensure humane treatment.

### 2.2. Chemicals and Reagents

*Polygonum multiflorum* Thunb. (Hebei Baihe Health Pharmaceutical Co., Ltd. (Baoding, China), Batch No.: 8220300801); Simvastatin Tablets (Jingxin Pharmaceutical Co., Ltd. (Shaoxing, China), Batch No.: A23032707); Basic feed (Ke’ao Xieli (Beijing, China)); High-fat feed (Ke’ao Xieli, Cat. No.: D12451). Sodium chloride (NaCl, Tianjin Zhiyuan Chemical Reagent Co., Ltd. (Tianjin, China)); Oil Red O Kit (Nanjing Jiancheng Bioengineering Institute (Nanjing, China), Cat. No.: D027-1-3); Trizol reagent (Yeasen Biotechnology Co., Ltd. (Shanghai, China), Cat. No.: 19202ES60); Green qPCR SuperMix, One-Step gDNA Removal and cDNA Synthesis SuperMix (TRAN (Beijing, China), Cat. No.: AQ601-02-V2, AT311-03); High-performance liquid chromatography (HPLC)-grade formic acid, acetonitrile, and formic acid were purchased from Sigma-Aldrich Co., Ltd. (St. Louis, MO, USA).

### 2.3. Preparation and Chemical Analysis of Polygonum multiflorum Extract

The medicinal materials of *Polygonum multiflorum* Thunb. were added with 8 times the volume of water, subjected to reflux extraction for 3 times, 1 h each time, and the filtrates were combined after filtration. The extract was concentrated to 1/5 of its original volume at 60–70 °C under −0.06~−0.08 MPa, centrifuged at 5000 rpm for 20 min to collect the supernatant, and further concentrated under reduced pressure to a clear ointment with a relative density of 1.25–1.28 (60 °C) for later use.

After pretreatment, the samples were qualitatively and quantitatively analyzed by ultra-high performance liquid chromatography-Orbitrap mass spectrometry (UPLC-Orbitrap MS). Chromatographic separation was performed on an Acquity T3 column (100 mm × 2.1 mm, 1.8 μm), with the mobile phase consisting of 0.05% formic acid aqueous solution (A) and acetonitrile (B). The gradient elution program was as follows: 0~1 min, 5~5% B; 1~33 min, 5~95% B; 33~35 min, 95~95% B; 35~36 min, 95~5% B; 36~40 min, 5~5% B. The flow rate was 0.3 mL/min, the column temperature was 40 °C, and the injection volume was 5 μL. Mass spectrometry was performed with an electrospray ionization (ESI) source, scanned separately in positive and negative ion modes, and the resolution of the Orbitrap mass analyzer was set to 70,000 FWHM (*m*/*z* 200) with a scanning range of *m*/*z* 70~1050. Compound Discoverer 3.0 software was used for identification according to mzCloud, mzVault, and OTCML databases, and relative quantification was performed using peak area.

### 2.4. Network Pharmacology Analysis

#### 2.4.1. Screening of Active Components and Targets

The active components of *Polygonum multiflorum* Thunb. were retrieved from the TCMSP, TCMID, and Herb databases, and screened and deduplicated with oral bioavailability (OB ≥ 30%) and drug-likeness (DL ≥ 0.18) as the criteria. The Smiles structures of the components were obtained from the PubChem database and imported into Swiss Target Prediction to predict the targets. Using “dyslipidemia” as the keyword, disease targets were collected from the Genecard and OMIM databases, standardized by Uniprot, and a Venn diagram was drawn using Venny 2.1 to obtain the common targets of *Polygonum multiflorum* Thunb. and dyslipidemia.

#### 2.4.2. Construction of PPI Network and Functional Enrichment Analysis

The common targets were imported into the String database to construct a protein–protein interaction (PPI) network. After removing isolated nodes, the network was imported into Cytoscape 3.9.1 for topological analysis. The DAVID database was used for Gene Ontology (GO) function and Kyoto Encyclopedia of Genes and Genomes (KEGG) pathway enrichment analysis, with the species set to “Homo sapiens”, and visual charts were drawn through the Microbioinformatics website.

### 2.5. Molecular Docking and Molecular Dynamics Simulation

#### 2.5.1. Molecular Docking Analysis (with PDB ID Annotation, Re-Docking Validation and Domain Definition)

To clarify the binding mode of 2,3,5,4′-tetrahydroxystilbene-2-O-β-D-glucoside (TSG, PubChem CID: 10200155, the main active component of *Polygoni Multiflori Radix*) with core pathway proteins, high-resolution (<2.5 Å) crystal structures were downloaded from the RCSB PDB database, and the binding domains were defined as follows: AMPK (PDB ID: 4CID, kinase domain of the α1 subunit, residues 30–280), LDLR (PDB ID: 1N7D, extracellular ligand-binding domain, residues 1–310), PCSK9 (PDB ID: 2P4E, catalytic domain residues 152–429 and C-terminal domain residues 430–692), and SREBP-2 (PDB ID: 6D2U, N-terminal DNA-binding domain residues 1–120 and regulatory domain residues 400–500). The 3D structure of TSG was constructed and energy-optimized using Chem3D 20.0 with the MMFF94 force field. Target proteins were preprocessed via PyMOL 2.5.5 to remove water molecules, ions, and original ligands, followed by hydrogen addition and side-chain optimization. Proteins and TSG were converted to PDBQT format with Gasteiger charges using ADFRsuite 1.0. Molecular docking was performed in AutoDock Vina 1.2.3 with a docking box of 20 × 20 × 20 Å centered on each functional domain, and the reliability was validated by re-docking (RMSD < 2.0 Å). The conformation with the lowest binding free energy was selected as the optimal binding mode, and visual analysis was conducted using PyMOL 2.5.5 and LigPlot+ v2.2 to characterize hydrogen bonds, hydrophobic interactions, π–π stacking, and salt bridges between TSG and key amino acid residues.

#### 2.5.2. Molecular Dynamics Simulation

Molecular dynamics simulation was performed in AMBER 24 software based on the initial structure of the complex obtained by docking. The small molecule was described by the GAFF2 force field, and the charge was calculated by the AM1-BCC method; the protein was described by the ff14SB force field. The system was solvated with the TIP3P water model, and K^+^/Cl^−^ was added to neutralize the charge. The simulation first performed 5000 steps of energy minimization, then heated to 298.15 K under the NVT ensemble and equilibrated for 500 ps, followed by 500 ps of NPT equilibration at 1 atm. Finally, a 100 ns NPT production simulation was carried out. In the simulation, a 10 Å non-bonded cutoff distance was set, the PME method was used to handle electrostatic interactions, the bond lengths of hydrogen atoms were constrained by the SHAKE algorithm, the temperature was controlled by Langevin dynamics (γ = 2 ps^−1^), the integration step size was 2 fs, and the trajectory was saved every 10 ps.

### 2.6. In Vivo Validation

#### 2.6.1. Model Establishment and Drug Administration

After 1 week of adaptive feeding, the mice were randomly divided into 6 groups (n = 6): normal feed diet (NFD) group (basic feed), high-fat diet (HFD) group (high-fat feed), HSW-L/M/H groups (high-fat feed + *Polygonum multiflorum* Thunb. extract 30/60/120 mg/kg), and simvastatin (SIM) group (high-fat feed + simvastatin 10 mg/kg). A dyslipidemia model was established by continuous feeding for 6 weeks. Subsequently, the HSW groups and SIM group were administered intragastrically, while the NFD group and HFD group were given an equal volume of distilled water, once a day for 28 consecutive days.

#### 2.6.2. Sample Collection and Index Detection

After the end of drug administration, the mice were fasted for 12 h, weighed, and blood was collected from the orbital venous plexus. Serum was separated by centrifugation, and the levels of triglycerides (TG), total cholesterol (TC), low-density lipoprotein cholesterol (LDL-c), high-density lipoprotein cholesterol (HDL-c), alkaline phosphatase (ALP), alanine aminotransferase (ALT), aspartate aminotransferase (AST), malondialdehyde (MDA), superoxide dismutase (SOD), and glutathione (GSH) were detected according to the kit instructions (Elabscience Biotechnology Co., Ltd., Wuhan, China; Cat. No.: E-BC-K261-M, E-BC-K109-M, E-BC-K205-M, E-BC-K221-M, E-BC-K091-M, E-BC-K235-M, E-BC-K236-M, E-BC-K025-M, E-BC-K022-M, E-BC-K030-M). The liver was dissected and weighed to calculate the liver index (liver weight/body weight × 100%). Part of the liver tissue was fixed in 4% paraformaldehyde for hematoxylin-eosin (HE) staining and Oil Red O staining; the remaining liver tissue was stored at −80 °C for reverse transcription-quantitative polymerase chain reaction (RT-qPCR) and Western blot detection.

#### 2.6.3. Histopathological Detection of Liver Tissue

After dehydration, embedding, and sectioning of the liver tissue, HE staining was performed to observe hepatocyte morphology, steatosis, and inflammatory infiltration; frozen sections were subjected to Oil Red O staining to quantitatively analyze the degree of lipid droplet accumulation.

#### 2.6.4. Detection of Gene Expression by RT-qPCR

Total RNA was extracted from liver tissue using Trizol reagent. After detecting RNA concentration and purity, reverse transcription was performed to synthesize complementary DNA (cDNA). With β-actin as the internal reference, the relative mRNA expression levels of AMPK, SREBP-2, PCSK9, and LDLR were calculated using the 2^−ΔΔCT^ method. The primer sequences are as follows (Table 1):

#### 2.6.5. Detection of Protein Expression by Western Blot

Total protein was extracted from the frozen liver tissue using a radioimmunoprecipitation assay (RIPA) lysis buffer supplemented with phenylmethylsulfonyl fluoride (PMSF) to prevent protein degradation. The protein concentration was determined using the bicinchoninic acid (BCA) protein assay kit. Equal amounts of protein samples were subjected to sodium dodecyl sulfate-polyacrylamide gel electrophoresis (SDS-PAGE) and then transferred onto polyvinylidene difluoride (PVDF) membranes. After transfer, the membranes were blocked with 5% non-fat milk in Tris-buffered saline with Tween 20 (TBST) for 1 h at room temperature to block non-specific binding sites. Subsequently, the membranes were incubated overnight at 4 °C with the following primary antibodies: AMPK and phosphorylated AMPK (p-AMPK) (CST, Cat. Nos.: 2532, 2535); SREBP-2, PCSK9, and LDLR (Proteintech, Cat. Nos.: 28212-1-AP, 27882-1-AP, 10785-1-AP); and β-actin (Affinity, Cat. No.: AF7018). After incubation with primary antibodies, the membranes were washed three times with TBST (10 min each wash) and then incubated with the corresponding horseradish peroxidase (HRP)-conjugated secondary antibody for 2 h at room temperature. Finally, the protein bands were visualized using an enhanced chemiluminescence (ECL) detection kit, and the gray value of each band was quantified using ImageJ 1.54p software. β-actin was used as the internal reference to normalize the protein expression levels.

### 2.7. Statistical Analysis

All experimental data were analyzed using GraphPad Prism 8.0 software and expressed as the mean ± standard deviation ($\overset{-}{x}$ ± SD). All data were first tested for normality via the Shapiro–Wilk test and for homogeneity of variance via Levene’s test. Differences between multiple groups were compared by one-way analysis of variance (one-way ANOVA) followed by Dunnett’s multiple comparison test. A *p*-value < 0.05 was considered statistically significant, and *p* < 0.01 was considered extremely statistically significant. The exact sample size for all experiments was n = 6, and the exact *p*-values were marked in the corresponding figures and text descriptions.

## 3. Results

### 3.1. Identification of HSW Components

**Figure 2:**
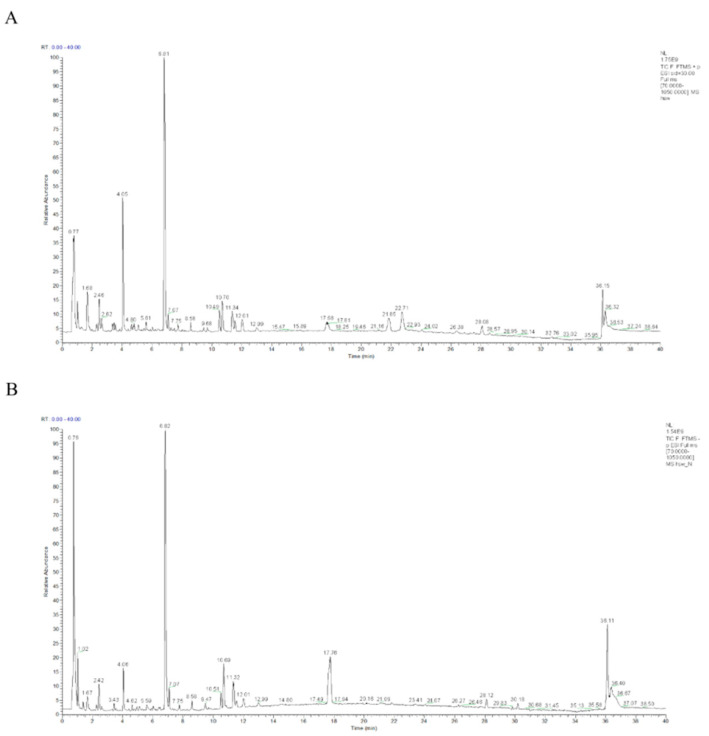
Total Ion Chromatogram (TIC) analysis of HCE by ultra-high performance liquid chromatography-Orbitrap mass spectrometry (UPLC-Orbitrap MS) in positive ion mode (**A**) and negative ion mode (**B**).

A total of 128 compounds were identified, including 25 terpenoids, 32 flavonoids, 28 phenolic acids, 15 phenylpropanoids, 12 quinones, 8 alkaloids, and 8 other compounds, covering the main structural types of reported compounds in the Polygonum genus. The base peak intensity (BPI) chromatograms of the *Polygonum multiflorum* Thunb. samples are shown in Figure 2 and Table 2.

### 3.2. Network Pharmacology Analysis

**Figure 3:**
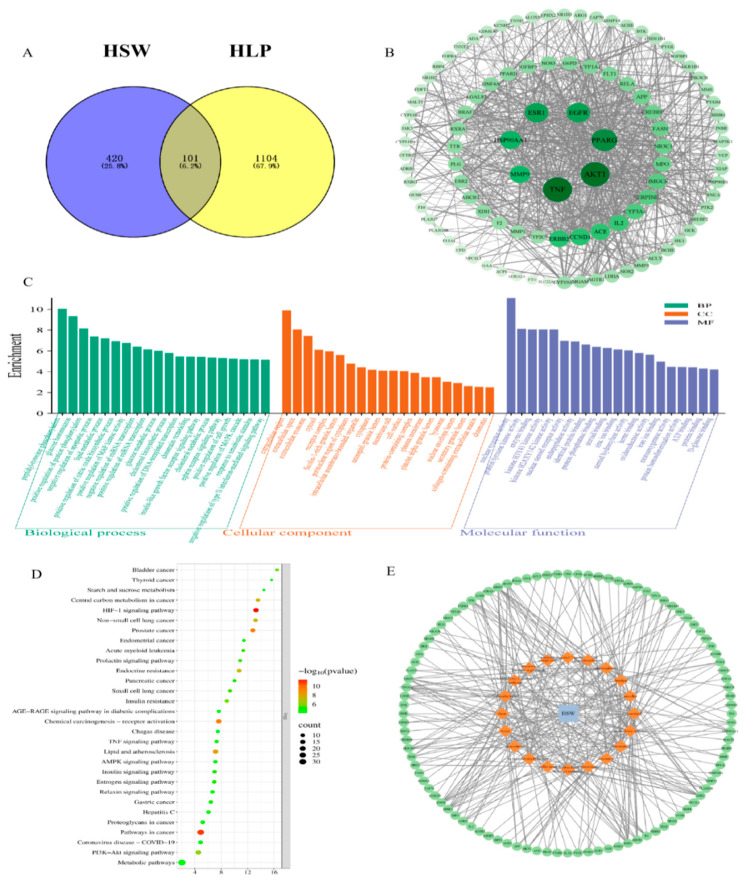
(**A**) Venn diagram of targets related to HSW and dyslipidemia (HLP), showing the intersection targets of HSW and HLP. (**B**) PPI network of HSW against HLP: among the 101 intersection targets, the darker the color of the target and the thicker the line, the greater the impact on HLP. (**C**) GO enrichment analysis: the *y*-axis represents fold enrichment, and the *x*-axis represents items; green, orange, and purple represent the 20 core results of biological process (BP), cellular component (CC), and molecular function (MF), respectively. (**D**) KEGG pathway enrichment analysis: the *y*-axis represents pathways, the *x*-axis represents false discovery rate (FDR), and the color change represents *p*-value; the size of the bubble indicates the number of enriched genes in the pathway. (**E**) “Herb-component-target” network diagram: HSW refers to *Polygonum multiflorum* Thunb.; orange-yellow rhombuses represent active components, and green circles represent protein targets.

Network pharmacology screening identified 22 active components and 521 related targets of *Polygonum multiflorum* Thunb., as well as 1205 disease targets of dyslipidemia. A total of 101 common targets were obtained from the intersection of the two groups (Figure 3A). Protein–protein interaction (PPI) network analysis showed that AKT1, TNF, PPARG, and other molecules were the core targets (Figure 3B). Gene Ontology (GO) functional enrichment analysis yielded 294 entries: biological processes mainly involved the regulation of lipid metabolism and cellular biological regulation; cellular components were dominated by vesicles and membrane rafts; molecular functions included nuclear receptor activity and signal receptor binding (Figure 3C). Kyoto Encyclopedia of Genes and Genomes (KEGG) pathway enrichment analysis screened out 134 pathways, and the core pathways included the AMPK signaling pathway, PI3K/AKT signaling pathway, and insulin resistance (Figure 3D). The “herb-component-target” network indicated that the active components exerted their effects through multiple targets and multiple pathways (Figure 3E).

### 3.3. Results of Molecular Docking and Molecular Dynamics Simulation

#### 3.3.1. Molecular Docking of Stilbene Glycoside with Core Target Proteins of Dyslipidemia

**Figure 4:**
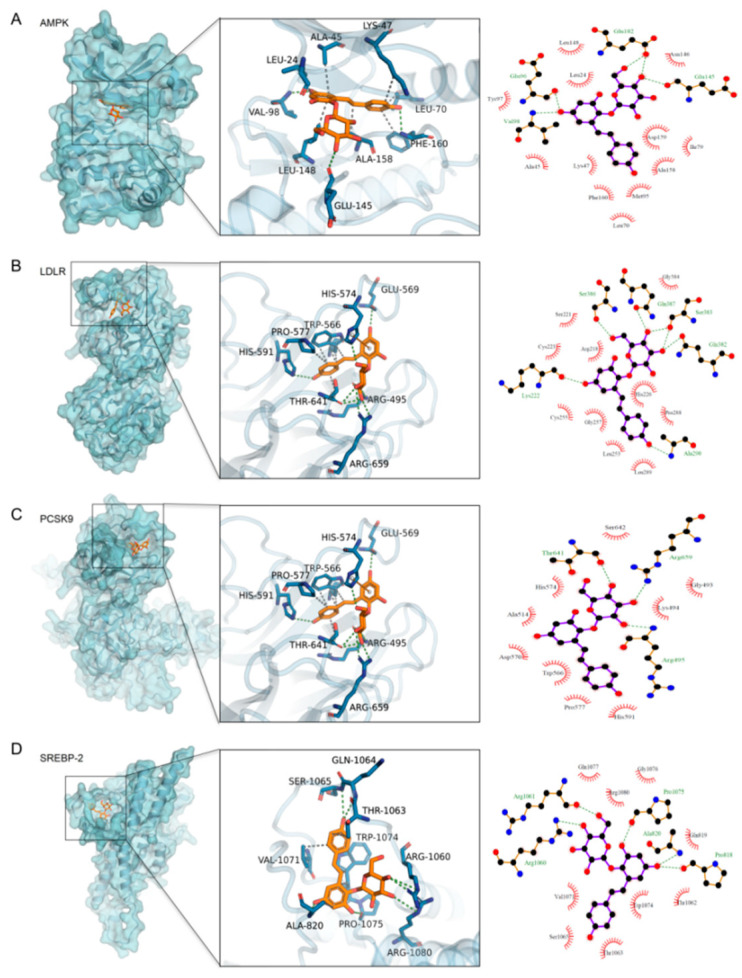
Ligand-binding structures of AMPK, LDLR, PCSK9, and SREBP-2: pocket localization and residue interactions (**A**–**D**). For each protein: the overall structure ((**left**) binding pocket framed), the local diagram of ligand-residue interactions ((**middle**) dashed lines indicate polar interactions), and the two-dimensional interaction schematic diagram (**right**).

The molecular docking results (see Figure 4) showed that stilbene glycoside had strong binding abilities with four target proteins, namely AMPK, SREBP-2, PCSK9, and LDLR, with binding free energies of −8.7, −9.2, −8.3, and −7.9 kcal/mol, respectively. Specifically, stilbene glycoside formed hydrogen bonds with Arg83 of AMPK and hydrophobic interactions with Ile124/Val136; it formed hydrogen bonds with Asn410 of SREBP-2 and hydrophobic contacts with Leu365/Phe366; in PCSK9, it formed hydrogen bonds with Ser153/Asp186 and embedded into the hydrophobic pocket composed of Phe379/Ile369; while its binding with LDLR mainly relied on π-hydrophobic interactions with Trp415/Pro432. The optimal docking conformations of all targets were located within the known functional pockets and were selected as the initial structures for subsequent molecular dynamics simulations.

#### 3.3.2. Molecular Dynamics Simulation of Core Targets

**Figure 5:**
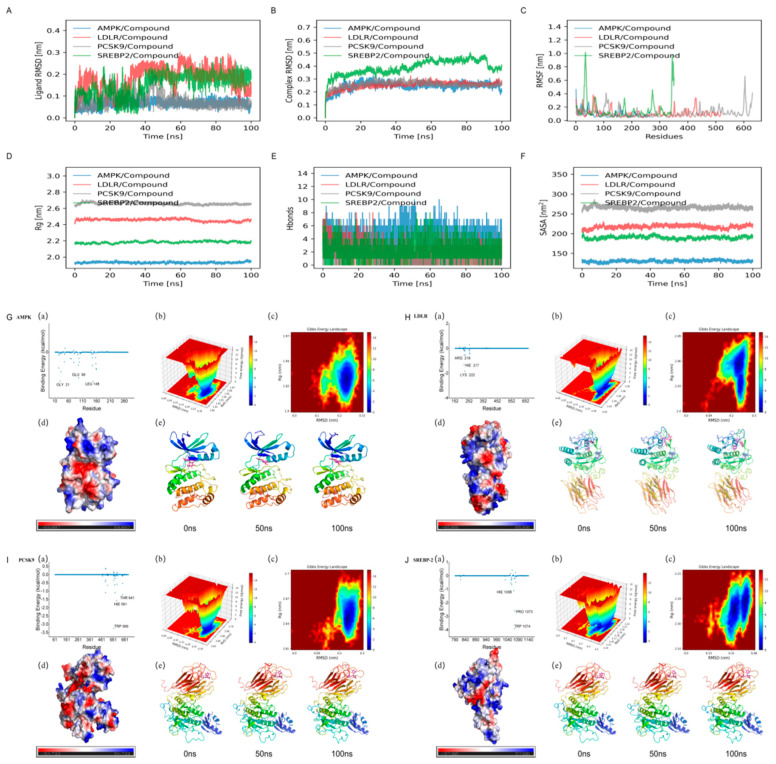
Molecular dynamics simulation characteristics of the compound with AMPK, LDLR, PCSK9, and SREBP-2 target proteins. (**A**) Changes in ligand root mean square deviation (RMSD) over simulation time; (**B**) Overall root mean square deviation of target-compound complexes; (**C**) Root mean square fluctuation (RMSF) of target protein residues; (**D**) Radius of gyration of the complexes; (**E**) Fluctuations in the number of hydrogen bonds between targets and the compound; (**F**) Solvent-accessible surface area of the complexes; (**G**–**J**) Represent fluctuations in binding free energy, residue fluctuation heat maps, protein surface flexibility heat maps, and conformational changes at 0 ns, 50 ns, and 100 ns of simulation for the complexes of PCSK9, HMGCR, LDLR, and SREBP-2 with the compound, respectively.

To further clarify the binding affinity between the compound (stilbene glycoside) and the core targets (AMPK, LDLR, PCSK9, and SREBP-2), molecular dynamics (MD) simulations were performed on the compound-target systems. The root mean square deviation (RMSD) was used to evaluate the conformational stability of the protein-ligand complexes and the degree of atomic position deviation. Generally, a lower RMSD value indicates that the complex is closer to the reference structure with stronger conformational stability, and the equilibrium state of the simulation system can be assessed by its changes. As shown in Figure 5A–C, all complexes reached equilibrium after 20 ns: among them, the LDLR-compound complex exhibited the smallest RMSD fluctuation range, while the SREBP-2-compound complex showed relatively larger fluctuations. If the radius of gyration (Rg) remains stable throughout the simulation, it indicates that the folded state of the protein structure is stable; the solvent-accessible surface area (SASA) is used to measure the proportion of the protein surface exposed to the aqueous phase, which can assist in predicting conformational changes during the interaction process. Further analysis showed that during the 100 ns simulation, the Rg values of the AMPK-compound, LDLR-compound, PCSK9-compound, and SREBP-2-compound complexes all maintained stable fluctuations, and no significant changes were observed in SASA (see Figure 5E,F). These results indicate that the compound did not cause obvious structural expansion or contraction of these complexes. The root mean square fluctuation (RMSF) can reflect the flexibility of amino acid residues in the protein. As shown in Figure 5D, the RMSF values of most complexes were lower than 3 Å, with only slight fluctuations in the local terminal loop regions, suggesting that compound binding did not disrupt the rigidity of the overall protein structure. Analysis of the binding pocket and conformational evolution showed that the LDLR-compound complex had the highest binding free energy, with the compound concentrated in the binding pocket and sustained stable interactions with key residues; the binding of AMPK and PCSK9 to the compound was also stable but with slightly lower affinity; while the SREBP-2-compound complex had the lowest binding free energy, with the compound distributed more dispersedly in the binding pocket and greater conformational flexibility (see Figure 5G–J). Collectively, these results demonstrate that the compound can form stable bindings with AMPK, LDLR, PCSK9, and SREBP-2, among which the binding stability and affinity with LDLR are the most prominent.

### 3.4. Effects of Polygonum multiflorum Thunb. on Body Weight and Blood Lipid Levels in Hyperlipidemic Mice

**Figure 6:**
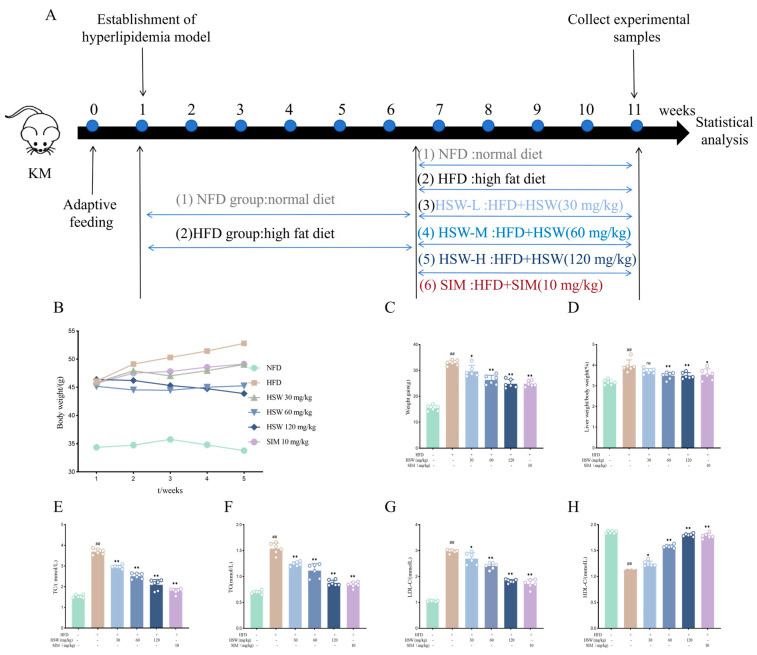
Effects of HSW on body weight gain and blood lipids in hyperlipidemic mice (n = 6). (**A**) Time schedule of model establishment and drug administration throughout the entire experimental process; (**B**) Changes in body weight of mice in each group during 4 weeks of treatment with *Polygonum multiflorum* Thunb. (HSW) or simvastatin (SIM); (**C**) Body weight gain of mice in each group; (**D**) Liver weight/body weight ratio; (**E**–**H**) Serum levels of TC, TG, LDL-c, and HDL-c in mice. Data are expressed as the mean ± standard deviation ($\overset{-}{x}$ ± SD) and analyzed by one-way analysis of variance (one-way ANOVA) followed by Dunnett’s test. Compared with the NFD group, ## *p* < 0.01; compared with the HFD group, * *p* < 0.05, ** *p* < 0.01. ns: no significance.

During the experiment, the time schedule for mouse model establishment, drug administration, sample collection, and other procedures is shown in Figure 6A. The body weight of mice in the HFD group was significantly higher than that in the control group (NFD group). However, after HSW administration, the body weight and liver index of mice decreased in a dose-dependent manner, and there was no significant difference between the HSW-H group and the SIM group (see Figure 6B–D). Compared with the normal group, the serum levels of total cholesterol (TC), triglycerides (TG), and low-density lipoprotein cholesterol (LDL-c) in the HFD group were increased, while the serum level of high-density lipoprotein cholesterol (HDL-c) was decreased. Nevertheless, HSW administration reversed this phenomenon, and the serum levels of TC, TG, LDL-c, and HDL-c changed with the dose of HSW, showing a significant dose-dependent effect (see Figure 6E–H). These results indicate that HSW exerts a good therapeutic effect on the blood lipid levels of HFD-induced hyperlipidemic mice.

### 3.5. Effects of Polygonum multiflorum Thunb. on Hepatic Histopathology and Liver Function in Hyperlipidemic Mice

**Figure 7:**
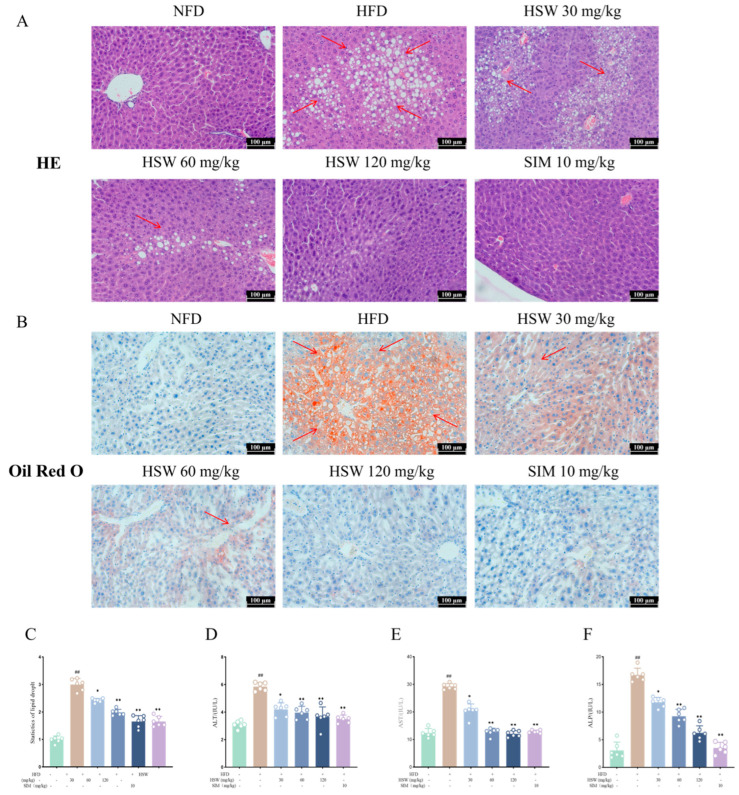
Effects of *Polygonum multiflorum* Thunb. (HSW) on hepatic pathology and liver function indices in hyperlipidemic mice induced by high-fat diet (HFD). (**A**) Hematoxylin-eosin (HE) staining results of liver tissue (Scale bar: 100 μm): red arrows indicate lipid droplet accumulation; NFD is the normal feed diet group, SIM is the simvastatin (10 mg/kg) positive control group, and HSW is set at 30, 60, and 120 mg/kg dose groups. (**B**) Oil Red O staining results of liver tissue (Scale bar: 100 μm): red staining represents lipid deposition, and red arrows indicate positive staining areas. (**C**–**F**) Serum biochemical indices: (**C**) is the serum alanine aminotransferase (ALT) level, (**D**) is the serum aspartate aminotransferase (AST) level, (**E**) is the serum triglyceride (TG) level, (**F**) is the serum total cholesterol (TC) level. Data are expressed as the mean ± standard deviation ($\overset{-}{x}$ ± SD); compared with the NFD group, ## *p* < 0.01; compared with the HFD group, * *p* < 0.05, ** *p* < 0.01.

Hematoxylin-eosin (HE) staining showed that hepatocytes in the NFD group had regular morphology with no lipid droplets; in contrast, hepatocytes in the HFD group were swollen, with a large number of lipid vacuoles, disordered arrangement, and inflammatory cell infiltration. In each HSW-treated group, the number of lipid vacuoles was reduced, and the arrangement of hepatocytes tended to be regular (Figure 7A). Oil Red O staining and quantitative analysis revealed that lipid droplet accumulation in liver tissue was significantly increased in the HFD group, while HSW administration reduced the number of lipid droplets in a dose-dependent manner (Figure 7B,C). Compared with the HFD group, the serum levels of alanine aminotransferase (ALT), aspartate aminotransferase (AST), and alkaline phosphatase (ALP) in each HSW-treated group were significantly decreased (*p* < 0.05 or *p* < 0.01) (Figure 7D–F), suggesting that HSW can alleviate HFD-induced liver injury.

### 3.6. Effects of Polygonum multiflorum Thunb. on Oxidative Stress Levels in Hyperlipidemic Mice

**Figure 8:**
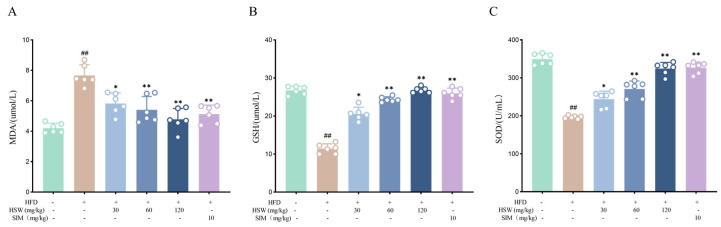
Effects of *Polygonum multiflorum* Thunb. (HSW) on oxidative stress indices in hyperlipidemic mice induced by high-fat diet (HFD). (**A**) Serum malondialdehyde (MDA) level; (**B**) Serum glutathione (GSH) level; (**C**) Serum superoxide dismutase (SOD) activity. Data are expressed as the mean ± standard deviation ($\overset{-}{x}$ ± SD); compared with the normal control group (NFD group), ## *p* < 0.001; compared with the HFD group, * *p* < 0.05, ** *p* < 0.01.

Compared with the NFD group, the serum malondialdehyde (MDA) level in the HFD group was significantly increased, while the serum superoxide dismutase (SOD) and glutathione (GSH) levels were significantly decreased (*p* < 0.01). After HSW administration, the MDA level decreased in a dose-dependent manner, and the SOD and GSH levels were increased in a dose-dependent manner (*p* < 0.05 or *p* < 0.01). There was no significant difference between the HSW-H group and the SIM group (Figure 8A–C), indicating that HSW can alleviate the oxidative stress imbalance induced by dyslipidemia.

### 3.7. Effects of Polygonum multiflorum Thunb. on the AMPK/SREBP-2/PCSK9/LDLR Signaling Pathway

**Figure 9:**
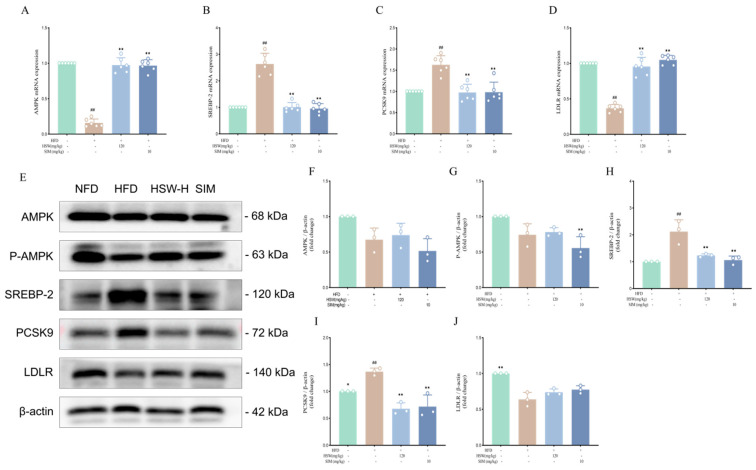
Regulatory effects of *Polygonum multiflorum* Thunb. (HSW) on lipid metabolism-related signaling pathway proteins in hyperlipidemic mice induced by a high-fat diet (HFD). (**A**–**D**). Relative mRNA expression levels of lipid metabolism-related genes: (**A**) is AMPK, (**B**) is SREBP-2, (**C**) is PCSK9, (**D**) is LDLR; HSW-H represents the HSW 120 mg/kg group, SIM is the simvastatin (10 mg/kg) positive control group, and NFD is the normal feed diet group. (**E**). Western blot detection results of lipid metabolism-related proteins: including AMPK, phosphorylated AMPK (P-AMPK), SREBP-2, PCSK9, LDLR, and internal reference β-actin. (**F**–**J**) Quantitative analysis of relative protein expression levels: (**F**) is AMPK, (**G**) is P-AMPK, (**H**) is SREBP-2, (**I**) is PCSK9, (**J**) is LDLR. Data are expressed as the mean ± standard deviation ($\overset{-}{x}$ ± SD); compared with the NFD group, ## *p* < 0.01; compared with the HFD group, * *p* < 0.05, ** *p* < 0.01.

Reverse transcription-quantitative polymerase chain reaction (RT-qPCR) results showed that compared with the NFD group, the mRNA expressions of AMPK and LDLR in liver tissue of the HFD group were significantly decreased, while the mRNA expressions of SREBP-2 and PCSK9 were significantly increased (*p* < 0.01). After HSW administration, the mRNA expressions of AMPK and LDLR were up-regulated, and the mRNA expressions of SREBP-2 and PCSK9 were down-regulated (*p* < 0.05 or *p* < 0.01) (Figure 9A–D). Western blot results demonstrated that the phosphorylation level of AMPK and the protein expression of LDLR in the HFD group were significantly decreased, while the protein expressions of SREBP-2 and PCSK9 were significantly increased (*p* < 0.01). After HSW intervention, the phosphorylation level of AMPK and the protein expression of LDLR were significantly increased, and the protein expressions of SREBP-2 and PCSK9 were significantly decreased (*p* < 0.05 or *p* < 0.01) (Figure 9E–J).

## 4. Discussion

Dyslipidemia and oxidative stress are potential inducements for the pathogenesis of cardiovascular diseases [22]. Dyslipidemia refers to high blood lipid levels, which can directly or indirectly aggravate the occurrence of cardiovascular system diseases. The main components of blood lipids are triglycerides and cholesterol, so dyslipidemia mainly refers to excessively high levels of triglycerides and cholesterol in the blood [23]. Therefore, reducing or controlling lipid accumulation and oxidative stress levels is an important approach to prevent dyslipidemia. This study found that HSW has the effects of anti-dyslipidemia and preventing atherosclerotic plaque formation; at the same time, it can also improve excessive fat accumulation, regulate blood lipid levels, improve liver function, and alleviate liver injury in HLP mice induced by high-fat feed [24].

AMPK acts as a master switch in regulating the balance of energy metabolism in the body, thus becoming a new target for the treatment of diseases such as obesity. The SREBP-2/PCSK9/LDLR signaling pathway regulates the expression of genes related to lipid synthesis and uptake. In previous studies, the SREBP-2/PCSK9 signaling pathway played an important role in cardiovascular diseases [25]. At the same time, relevant studies have shown that the expression of SREBP-2 is significantly increased in obese rats fed a high-fat diet [26]; overexpression of SREBP-2 can significantly increase lipid deposition and the expression of genes related to lipid synthesis and metabolism [27]. Jeong HJ et al. found an SRE binding site of SREBP-2 in the promoter region of protein convertase subtilisin/kexin type 9 (PCSK9), and showed that SREBP-2 can enhance the promoter activity of PCSK9 [28]. PCSK9 is a serine protease with high expression in the liver. It can promote cholesterol homeostasis by regulating the level of low-density lipoprotein receptor (LDLR) in the liver [29,30]. This study showed that in the liver of hyperlipidemic mice induced by a high-fat diet, the mRNA expression levels of AMPK and LDLR were decreased, and the mRNA expressions of PCSK9 and SREBP-2 were increased, while HSW could reverse these situations. Therefore, this study indicates that HSW can regulate AMPK and its downstream SREBP-2/PCSK9/LDLR signaling pathway, and this mechanism has been verified in in vitro experiments. This reveals that HSW provides strong scientific evidence for the prevention and treatment of dyslipidemia and the reduction in lipid accumulation.

Cardiovascular system diseases such as dyslipidemia and type 2 diabetes are all related to lipid accumulation in tissues [31,32,33]. Therefore, alleviating lipid accumulation is an effective strategy to prevent these diseases. In the process of lipid accumulation, excessive oxidative stress will lead to the massive production of ROS, which in turn regulates antioxidant factors (SOD, MDA, GSH, etc.) to actively respond to the antioxidant mechanism, thereby minimizing the damage caused by excessive oxidative stress [34,35]. The results of this study showed that after HSW treatment, the level of MDA in the liver tissue of hyperlipidemic mice was significantly decreased, while the levels of GSH and SOD were significantly increased. It indicates that HSW alleviates the degree of oxidative damage, improves the antioxidant capacity, and ameliorates the abnormal lipid metabolism in hyperlipidemic mice.

In summary, this study confirms that HSW can effectively improve the blood lipid levels of HFD-induced hyperlipidemic mice, alleviate hepatic lipid accumulation and liver injury. Its lipid-lowering effect is closely related to regulating the AMPK/SREBP-2/PCSK9/LDLR signaling pathway, alleviating oxidative stress, and reducing fat deposition. These findings not only provide a new perspective for explaining the lipid-lowering mechanism of HSW but also lay a scientific basis for its clinical application. However, this study still has certain limitations: the main active components in HSW and their individual effects have not been clarified; secondly, there is a complex regulatory network downstream of AMPK, while this study only focuses on the SREBP-2/PCSK9/LDLR pathway, and other potential targets need to be further explored.

To address the limitations of this study and promote the in-depth research and practical application of HSW, future research plans will focus on the following aspects: First, we will further verify the causal relationship of the AMPK/SREBP-2/PCSK9/LDLR pathway in hepatocyte models, and clarify the direct action targets of the active components of HSW. Second, we will optimize the preparation process of HSW extract, purify the key active components, and explore their individual and synergistic effects to clarify the material basis of the lipid-lowering effect of HSW. Third, long-term toxicity experiments and clinical preliminary trials will be carried out to systematically evaluate the safety and clinical applicability of HSW extract, providing a solid basis for its subsequent clinical application. In terms of clinical transformation, the results of this study provide scientific evidence for the application of HSW in the prevention and adjuvant treatment of human dyslipidemia and related fatty liver diseases. In the future, we can further develop natural drugs or dietary supplements based on the active components of HSW, which is expected to provide new ideas and schemes for the clinical treatment of metabolism-related diseases, and promote the transformation of basic research results into clinical practical value.

## 5. Conclusions

This study systematically elucidated the molecular mechanism by which Polygoni Multiflori Radix (PMR) ameliorates high-fat diet (HFD)-induced dyslipidemia in mice, combining network pharmacology, molecular docking, molecular dynamics simulation, and in vivo animal experiments. Network pharmacology screening identified 22 active components and 101 common targets of PMR against dyslipidemia, with the AMPK sig-naling pathway confirmed as the core regulatory pathway. Molecular docking and 100 ns molecular dynamics simulation further verified that 2,3,5,4′-tetrahydroxystilbene-2-O-β-D-glucoside (TSG), the main active component of PMR, forms stable binding interactions with the key proteins of the AMPK/SREBP-2/PCSK9/LDLR axis (AMPK, SREBP-2, PCSK9, LDLR), with the most prominent binding affinity and stability observed for LDLR.

In vivo experiments demonstrated that PMR extract exerts a dose-dependent li-pid-lowering effect in HFD-induced hyperlipidemic mice: it significantly reduces body weight and liver index, reverses the abnormal elevation of serum total cholesterol (TC), tri-glycerides (TG), and low-density lipoprotein cholesterol (LDL-c), and restores the de-creased level of high-density lipoprotein cholesterol (HDL-c). Histopathological analysis confirmed that PMR alleviates HFD-induced hepatic steatosis, inflammatory cell infiltra-tion, and lipid droplet accumulation, while also reducing the serum levels of alanine aminotransferase (ALT), aspartate aminotransferase (AST), and alkaline phosphatase (ALP), thereby ameliorating liver injury. Additionally, PMR effectively regulates oxidative stress balance in hyperlipidemic mice, as evidenced by the reduced malondialdehyde (MDA) level and elevated superoxide dismutase (SOD) and glutathione (GSH) levels in serum.

At the molecular level, PMR upregulates the mRNA and protein expression (includ-ing phosphorylation) of AMPK in mouse liver tissue, downregulates the transcriptional and translational levels of SREBP-2 and PCSK9, and increases the expression of LDLR. This regulatory effect on the AMPK/SREBP-2/PCSK9/LDLR signaling pathway enhances hepatocellular uptake of serum LDL-c, improves hepatic lipid metabolism disorder, and ultimately exerts an anti-dyslipidemic effect. Collectively, these results confirm that PMR improves dyslipidemia by activating the AMPK pathway, inhibiting the downstream SREBP-2/PCSK9 cascade, stabilizing LDLR expression, and simultaneously alleviating oxidative stress and hepatic lipid accumulation.

This study clarifies the material basis and molecular mechanism of the lipid-lowering effect of PMR, filling the research gap in the systematic exploration of its overall regulatory mechanism on lipid metabolism-related signaling pathways, and providing sufficient scientific evidence for the clinical application of PMR in the prevention and treatment of dyslipidemia and its associated fatty liver disease. Although the study has certain limita-tions (e.g., the individual effects of PMR’s active components and the exploration of other downstream targets of AMPK remain to be in-depth), it lays a foundation for subsequent research on the purification of PMR’s key active components, verification of their synergis-tic effects, and long-term safety evaluation. In the context of the clinical limitations of tra-ditional lipid-lowering drugs (e.g., statins with potential side effects), PMR, as a natural traditional Chinese medicine, shows great potential for development as a safe and effective lipid-lowering agent or dietary supplement, providing a new alternative for the clinical management of dyslipidemia and metabolic diseases associated with lipid metabolism disorders.

## Figures and Tables

**Table 1:** The primer sequences.

Gene	Forward Primer (5′ → 3′)	Reverse Primer (5′ → 3′)
AMPK	AGGAATTCAATGACGTGTACCT	AGGTCCCTGTGAATTATGTCAG
SREBP-2	TGAAGCTGGCCAATCAGAAAA	CCACATCACTGTCCACCAGACT
PCSK9	CCTGCCTTTGTGGTGAAGATGA	TGACCCTGCCCTCAATTTCC
LDLR	TGACTCAGACGAACAAGGCTG	ATCTAGGCAATCTCGGTCTCC
β-actin	CACCATGTACCCAGGCATTG	CCTGCTTGCTGATCCACATC

**Table 2:** Table of Identified Compounds from *Polygonum multiflorum* (LC-MS, POS Mode).

Compound Name	RT [min]	Exact *m*/*z*	Adduct Type	MS/MS Fragments (Key Ions)	Confidence Level ^1^	Relative Abundance ^2^
(-)-Epicatechin	6.8	291.0861	[M+H]^+^	273.0755, 245.0806	High (98)	3.25 × 10^6^
(R)-Sulcatol	24	111.1172	[M+H-H_2_O]^+^	83.0905, 69.0749	Low (11.3)	5.29 × 10^6^
1,6-ANHYDRO-B-GLUCOSE	10.7	145.0496	[M+H-H_2_O]^+^	127.0390, 109.0284	Low (13.2)	2.93 × 10^6^
1-Acetylimidazole	1	262.1284	[M+Na]^+^	240.1078, 110.0481	Medium (23.7)	1.61 × 10^6^
1-AMINOCYCLOPROPANE-1-CARBOXYLATE	0.8	85.029	[M+H-CO_2_]^+^	68.0023, 56.0125	High (67.1)	4.89 × 10^7^
1-AMINOCYCLOPROPANE-1-CARBOXYLATE	1	84.0451	[M-H_2_O+H]^+^	67.0184, 55.0286	Medium (48.9)	1.29 × 10^7^
1-Hydroxyanthraquinone	11.4	225.0544	[M+H]^+^	207.0438, 181.0540	Medium (48.3)	1.16 × 10^7^
1-Methylxanthine	1	167.0555	[M+H]^+^	149.0449, 123.0551	Medium (53.9)	1.97 × 10^6^
1-Naphthol	8.6	145.0648	[M+H]^+^	127.0542, 115.0543	High (90.9)	2.73 × 10^6^
1-OLEOYL-RAC-GLYCEROL	22.4	339.2894	[M+H-H_2_O]^+^	281.2528, 253.2630	Medium (62.4)	4.03 × 10^6^
Acetophenone	6.8	121.065	[M+H]^+^	105.0701, 77.0599	High (78.8)	2.36 × 10^8^
Acetophenone	7.1	121.065	[M+H]^+^	105.0700, 77.0598	Medium (51.0)	6.27 × 10^6^
Adenine	0.8	136.0617	[M+H]^+^	119.0350, 93.0452	High (95.8)	3.00 × 10^7^
Adenine	1	136.0619	[M+H]^+^	119.0352, 93.0453	High (91.2)	4.37 × 10^7^
Apigenin	10.3	271.0601	[M+H]^+^	253.0495, 225.0547	High (94.3)	2.91 × 10^6^
Apigenin	10.7	271.0599	[M+H]^+^	253.0493, 225.0545	High (97.7)	6.29 × 10^8^
Choline	0.7	104.1074	[M+H]^+^	87.0807, 60.0709	High (94.4)	1.19 × 10^8^
Gallic acid	7.1	153.0181	[M+H-CO_2_]^+^	125.0283, 109.0385	High (82.5)	1.53 × 10^8^
Genistein	9.8	271.0599	[M+H]^+^	253.0493, 225.0545	High (97.7)	1.82 × 10^7^
Glycitein	12	285.0754	[M+H]^+^	267.0648, 239.0700	High (98.5)	2.28 × 10^8^
Guanine	1.1	152.0567	[M+H]^+^	135.0300, 109.0402	High (92.9)	1.71 × 10^7^
L-Arginine	0.8	175.1189	[M+H]^+^	158.0922, 130.1024	High (90.1)	6.28 × 10^7^
L-Tyrosine	0.8	182.0813	[M+H]^+^	165.0546, 137.0648	High (95.7)	6.61 × 10^6^
Resveratrol	6.6	229.0859	[M+H]^+^	211.0753, 185.0855	High (94.8)	2.31 × 10^6^
Umbelliferone	6.5	163.039	[M+H]^+^	135.0492, 107.0544	Medium (73.2)	3.57 × 10^6^
Umbelliferone	6.8	163.0389	[M+H]^+^	135.0491, 107.0543	High (94.1)	1.32 × 10^7^

## Data Availability

The original contributions presented in this study are included in the article. Further inquiries can be directed to the corresponding author.
